# Supplementary material for: Quantitative Virus-Associated RNA Detection to Monitor Oncolytic Adenovirus Replication
Source: Int J Mol Sci. 2024 Jun 14;25(12):6551. doi: 10.3390/ijms25126551 (PMC11203762; doi:10.3390/ijms25126551)
Supplement: Supplementary file 1 [file ijms-25-06551-s001.zip › ijms-3034462-supplementary.pdf]

Supplemental data

Table S1: Sequences of primers used to detect adenovirus VA-RNA species. Each reverse transcription SL primer anneals to the last six nucleotides of the VA-RNA sequence (yellow); each forward primer anneals to the 5’ 15 or 16 nucleotides of the VA-RNA (green) and was artificially extended with the TACGAT sequence (bold) to increase the annealing temperature; the universal reverse primer was designed to specifically anneal to the SL sequence on the reverse transcription primer (underlined *italics*) [21].

	NAME	SEQUENCE 5’-3’
VA RNAI-5p	**RNA sequence**	AGCGGGCACUCUUCCGUGGUCUG
	**SL primer**	GTCGTAT*CCAGTGCAGGGTCCGAGGTA*TTCGCACTGGATACGACCAGACC
	**Forward**	**TACGAT**AGCGGGCACTCTTCC
VA RNAI-3p	**RNA sequence**	GACAACGGGGGAGUGCUCCUU
	**SL primer**	GTCGTAT*CCAGTGCAGGGTCCGAGGTA*TTCGCACTGGATACGACAAGGAG
	**Forward**	**TACGAT**GACAACGGGGGAGTG
VA RNAII-3p	**RNA sequence**	UCCGGAAACAGGGACGAGCCCC
	**SL primer**	GTCGTAT*CCAGTGCAGGGTCCGAGGTA*TTCGCACTGGATACGACGGGGCT
	**Forward**	**TACGAT**TCCGGAAACAGGGACG
	**Universal reverse**	CCAGTGCAGGGTCCGAGGTA

```
5′-TACGATAGCGGGCACTCTTCC-3′                                FWD primer

        5′-AGCGGGCACUCUUCCGUGGUCUG-3′                      miVARNA I

                       SL primer  3′-CCAGACCAGCATAGGTCACGCTTATGGAGCCTGGGACGTGACCTATGCTG-5′
```

Figure S1: Graphical illustration of the RT-qPCR method to detect VA-RNAI-5p. Sequence of the 5′ end of VA-RNA I defining processed miVARNAI-5p according to Punga et al. [7] is shown in red, with the SL primer used in the RT step (recognition SL sequence for the PCR reverse primer underlined) below; and the PCR forward primer above the miVARNAI-5p template.

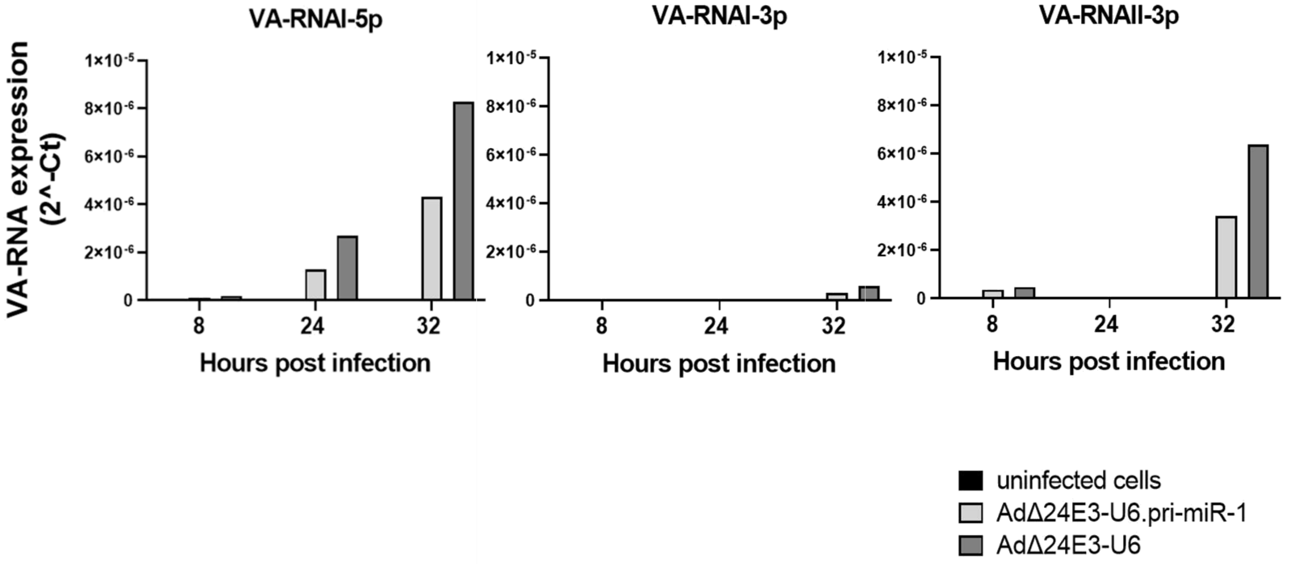

Figure S2: Comparison of primer sets designed for detection of different VA-RNA species. HCT116 cells were infected at MOI 100 with the indicated oncolytic adenoviruses and RT-qPCR was done on cell lysates after 8, 24 or 32 hours. VA-RNAI-5p was selected for further use because it could already be detected after 24 hours.

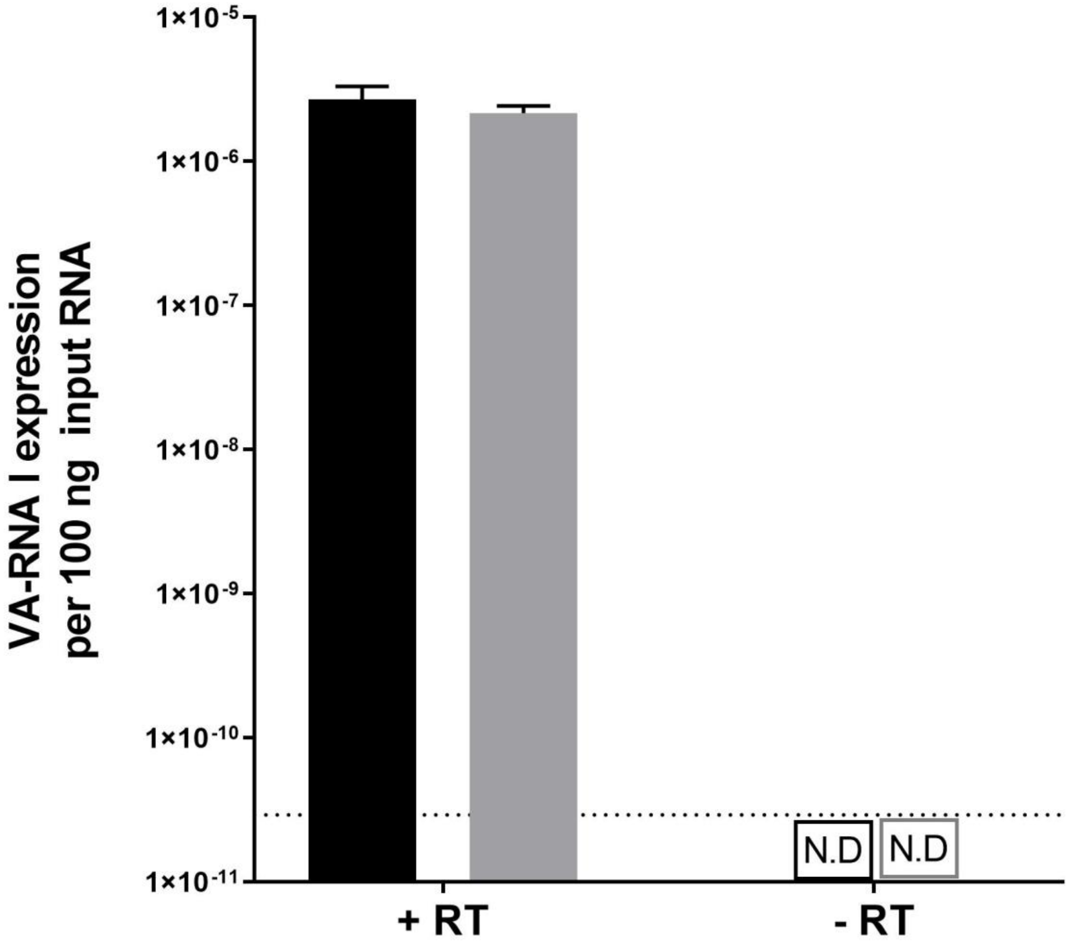

Figure S3: Specificity of the RT-qPCR assay for VA-RNAI-5p. RNA was isolated from A549 cells infected with Ad5 at MOI-100 IU/cell 32 hours earlier; and subjected to the RT-qPCR assay under standard conditions (black bar), or without reverse transcriptase in the cDNA preparation step (-RT) and/or after treating the isolated RNA with DNase I (grey bar). Data are means from two independent experiments. N.D., not detected; dotted line represents limit of detection.

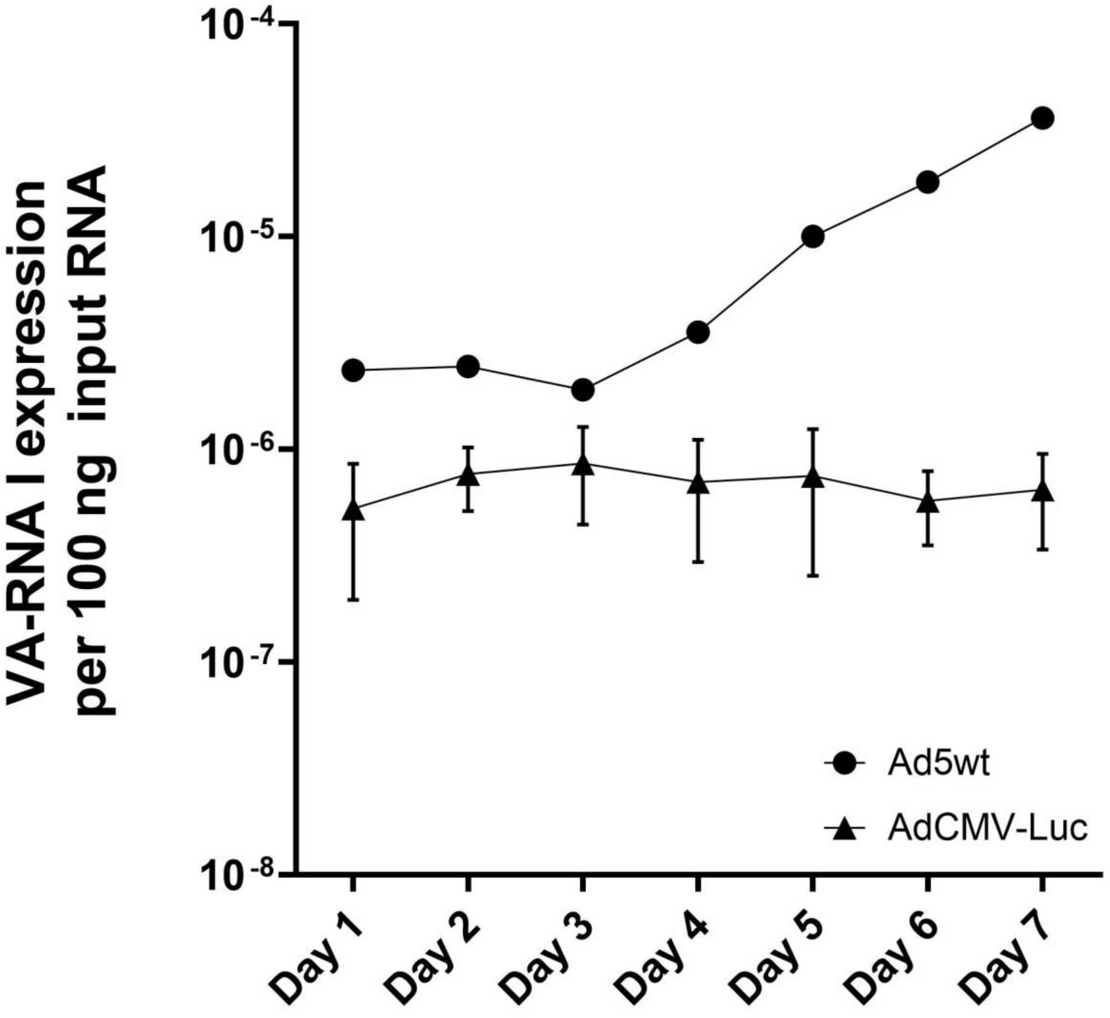

Figure S4: VA-RNA I expression in A549 cell cultures infected with Ad5 or AdCMV-Luc. Cells were infected at MOI 100 IU/cell and monitored over a 7-day period. Ad5 data are from a single experiment; AdCMV-Luc data are from three independent experiments and are shown as mean ± SD.
